# Supplementary material for: Characterization of SARS-CoV-2 and host entry factors distribution in a COVID-19 autopsy series
Source: Commun Med (Lond). 2021 Aug 23;1:24. doi: 10.1038/s43856-021-00025-z (PMC9053209; doi:10.1038/s43856-021-00025-z)

Characterization of SARS-CoV-2 and host entry factors distribution in a COVID-19 autopsy series

Xiao-Ming Wang[1, 2*], Rahul Mannan[1, 2*], Lanbo Xiao[1, 2], Eman Abdulfatah[1], Yuanyuan Qiao[1, 2], Carol Farver[1], Jeffrey L. Myers[1], Sylvia Zelenka-Wang[1, 2], Lisa McMurry[1], Fengyun Su[2], Rui Wang[2], Liron Pantanowitz[1], Jeffrey Jentzen[1], Allecia Wilson[1], Yuping Zhang[2], Xuhong Cao[2], Arul M. Chinnaiyan[1,2,3,4,5^], Rohit Mehra[1,2.3^]

[1]Department of Pathology, University of Michigan Medical School, Ann Arbor, MI, USA

[2]Michigan Center for Translational Pathology, Ann Arbor, MI, USA

[3]Rogel Cancer Center, Michigan Medicine, Ann Arbor, MI, USA

[4]Department of Urology, University of Michigan Medical School, Ann Arbor, MI, USA

[5]Howard Hughes Medical Institute, Ann Arbor, MI, USA

*X.W., and R. Mannan contributed equally to this work.

^A.M.C. and R. Mehra contributed equally to this work.

Supplementary Table 1. RNA-ISH target probe information.

Target Gene	Probe Name	Catalog Number from ACD	NCBI Reference Sequence (RefSeq) Accession	Start/End location (nucleotide)
SARS-CoV-2 S plus strand	V-nCoV2019-S	848561	NC_045512.2	21631 - 23303
SARS-CoV-2 S minus strand	V-nCoV2019-S-sense	845701	NC_045512.2 (minus strand)	21631 - 23303
TMPRSS2	Hs-TMPRSS2	470341	NM_001135099.1	459 - 1552
ACE2	Hs-ACE2	848151	NM_021804.3	307 - 1267
AR	Hs-AR	400491	NM_000044.3	5604 - 6660
SARS-CoV-2 N positive strand	V-SARS-CoV-2-N-O1-C2	863831-C2	MT020880.1	28274-28882

Supplementary Table 2. Clinicopathologic characteristics of the study cases in this autopsy cohort.

Case ID	Pulmonary histopathologic findings	Non-pulmonary histopathologic findings	Other significant conditions
1	Diffuse alveolar damage (DAD) with prominent hyaline membranes; lymphocytic infiltrate; hyperplastic alveolar pneumocytes	Heart: myofibrillary hypertrophy with pericardial chronic inflammatory reaction<br>Kidney: tubular epithelial sloughing admixed with red blood cells<br>Spleen: depletion of lymphocytes and red cells with viral nuclear smudging<br>Pancreas: diffuse autolysis	Type A aortic dissection-operated
2	Diffuse alveolar damage (DAD) with prominent hyaline membranes; lymphocytic infiltrate; hyperplastic alveolar pneumocytes; alveolar epithelial cytoplasmic vacuolation; coagulopathy	Heart: myofibrillary hypertrophy<br>Kidney: diffuse autolysis with proteinaceous material in Bowman's capsule<br>Liver: autolysis, mild to moderate steatosis<br>Spleen: autolysis<br>Pancreas: diffuse autolysis	Cardiomegaly, hepatomegaly hypertension, diabetes and obesity, acute renal failure
3	Diffuse alveolar damage (DAD) with prominent hyaline membranes; lymphocytic infiltrate; coagulopathy	Heart: myofibrillary hypertrophy with interstitial fibrosis<br>Native kidney: features of end-stage renal disease (ESRD)<br>Post-transplant kidney: thickening of vessels and a focal accumulation of large, discohesive cells with foamy cytoplasm and multinucleation in the medulla<br>Spleen: autolysis<br>Pancreas: autolysis<br>Uterus: cystic endometrial atrophy	Ischemic bowel disease, hypotension, diabetes
4	Diffuse alveolar damage (DAD) with prominent hyaline membranes; lymphocytic infiltrate; coagulopathy; features associated with underlying asthma-mucus plugging, goblet cell metaplasia, mucus gland hyperplasia, thickening of sub-epithelial basement membranes, and fibrinous bronchopneumonia	Heart: myofibrillary hypertrophy<br>Kidney: tubular calcifications<br>Liver: moderate steatosis and congestion	Asthma, hemopericardium, pleural effusions and ascites
5	Diffuse alveolar damage (DAD) with prominent hyaline membranes; lymphocytic infiltrate; acute and organizing bronchopneumonia, hemorrhage and edema; reactive changes and focal	Heart: multifocal areas of fibrosis, perivascular chronic inflammation, focal early ischemic changes in the right ventricle	Coronary artery disease, hypertension, type 2 diabetes mellitus

	areas of acute inflammation in the tracheobronchial tree		
6	Severe emphysematous changes, lymphocytic infiltrate; edema; coagulopathy (capillaritis); and bronchopneumonia	Heart: myofibrillary hypertrophy and patchy areas of interstitial fibrosis, chronic inflammation in the visceral pericardial surface	Pulmonary emphysema, medical history of diabetes mellitus II, stroke, hypertension and dementia

Supplementary Table 3. SARS-CoV-2 qRT-PCR and RNA-ISH result comparison.

Sample Info	RNA-ISH	qRT-PCR Ct value			
	SARS-CoV-2 S gene RNA-ISH Summary*	SARS-CoV-2 qRT-PCR summary#	SARS-CoV-2 E gene	SARS-CoV-2 N gene	SARS-CoV-2 RdRP gene
**Patient 1**					
Patient 1 Lung	+++	++++	19.4	18.2	19.5
Patient 1 Bronchus	+	+++	28.8	26.4	29.3
Patient 1 Kidney	-^	+	Undetermined	36.0	35.8
Patient 1 Heart	-^	++	28.7	27.4	28.9
Patient 1 Lymph node	+	++	31.8	29.7	30.8
Patient 1 Thyroid	-^	++	36.3	33.1	34.3
Patient 1 Spleen	-^	++	Undetermined	34.8	Undetermined
Patient 1 Prostate block 1	-^	++	Undetermined	36.0	33.3
Patient 1 Prostate block 2	-^	+	Undetermined	35.1	34.9
Patient 1 Colon	-^	++	34.0	34.2	35.9
**Patient 2**					
Patient 2 Lung	++	+++	29.1	27.1	27.1
Patient 2 Liver	-^	-	Undetermined	Undetermined	Undetermined
**Patient 3**					
Patient 3 Lung	++++	++++	20.1	19.4	17.7
Patient 3 Trachea	+	++	32.2	30.7	31.8
Patient 3 Trachea	+	++	32.1	30.4	32.2
Patient 3 Kidney	+	+	38.8	36.2	34.5
Patient 3 Liver	-^	+	Undetermined	37.2	Undetermined
Patient 3 Uterus	-^	++	Undetermined	33.8	34.0
Patient 3 Pancreas	-	-	Undetermined	Undetermined	Undetermined
**Patient 4**					
Patient 4 Lung	+++	+++	23.7	22.9	23.3
Patient 4 Bronchus	++	++	32.1	30.6	32.0
Patient 4 Heart, LV	-^	++	34.1	33.3	34.5
Patient 4 Heart, RV	-^	+	Undetermined	36.4	Undetermined
Patient 4 Adrenal	-	+	Undetermined	38.7	36.1
**Control Samples**					
Normal Lung	-	-	Undetermined	Undetermined	Undetermined
H1N1 Influenza Lung	-	-	Undetermined	Undetermined	Undetermined
Normal Prostate	-	-	Undetermined	Undetermined	Undetermined

*****For RNA-ISH data, ++++=very strong; +++=strong, ++=weak; +=very weak

#For qRT-PCR data, ++++=Ct<20; +++=Ct>20 <30; ++=Ct>30 <35; +=Ct>35; -=undetermined

^Rare signal clusters were observed, but no individual brown dot signals observed, counted as SARS-CoV-2 negative

Supplementary Table 4. SARS-CoV-2 strand-specific qRT-PCR for detection of viral replication or single-strand virus.

Sample Info	RNA-ISH		qRT-PCR Ct value		
	SARS-COV-2 S gene minus strand	SARS-COV-2 S gene plus strand	SARS-COV-2 E gene minus strand	SARS-COV-2 E gene plus strand	RNaseP control
Patient 1 Lung block 1	Positive	Positive	31.0	24.3	25.0
Patient 1 Lung block 2	Positive	Positive	25.1	20.5	23.9
Patient 3 Lung	Positive	Positive	26.1	20.1	25.7
Patient 3 Trachea	Not detected	Positive	Undetermined	30.9	27.0
Patient 4 Lung	Positive	Positive	33.6	25.8	27.0
Patient 5 Lung	Positive	Positive	32.5	26.6	25.9
Normal Lung	Not detected	Not detected	Undetermined	Undetermined	26.0
Water Blank	NA	NA	Undetermined	Undetermined	Undetermined

Supplementary Figure 1. Confirmation of RNA-ISH signal specificity of SARS-CoV-2-S and SARS-CoV-2-S-sense probes in lung tissues by RNase treatment. SARS-CoV-2 virus signals were detected by SARS-CoV-2-S RNA-ISH in the lung alveolar hyaline membranes and in the interstitial regions in Patient 1 (**a**) and Patient 3 (**b**), but not detected in tissue sections treated with RNase prior to target probe hybridization in Patient 1 (**c**) or Patient 3 (**d**). SARS-CoV-2 viral replication were detected by SARS-CoV-2-S sense RNA-ISH in lung tissues in Patient 1 (**e**) and Patient 3 (**f**), but not detected after RNase treatment in Patient 1 (**g**) or Patient 3 (**h**). Arrows point to RNAISH signals. No signals were detected by negative control DapB RNA-ISH (**i.** Patient 1 and **j.** Patient 3). Scale bars=100μm

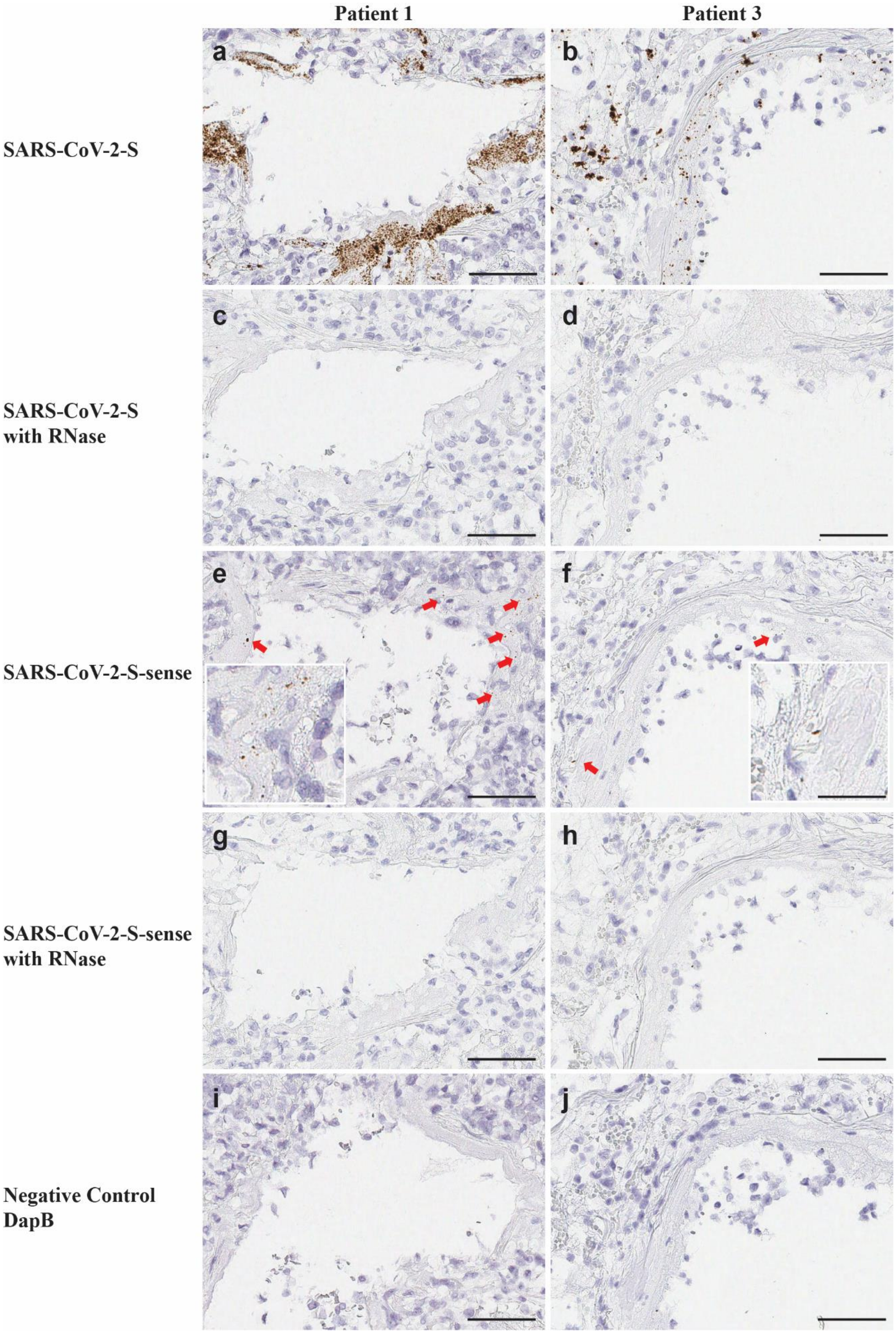
Patient 1
Patient 3
SARS-CoV-2-S
SARS-CoV-2-S with RNase
SARS-CoV-2-S-sense
SARS-CoV-2-S-sense with RNase
Negative Control DapB
a
b
c
d
e
f
g
h
i
j

Supplementary Figure 2. SARS-CoV-2 virus detection in pulmonary parenchyma by immunohistochemistry (IHC). SARS-CoV-2 viruses were detected by nucleocapsid antibody and S RNA-ISH probe within the lung alveolar hyaline membranes (**a** & **b**) and intra-alveolar septa (**c** & **d**). No signals were observed in normal lung tissues from uninfected individual (**e & f**). Scale bars=200μm.

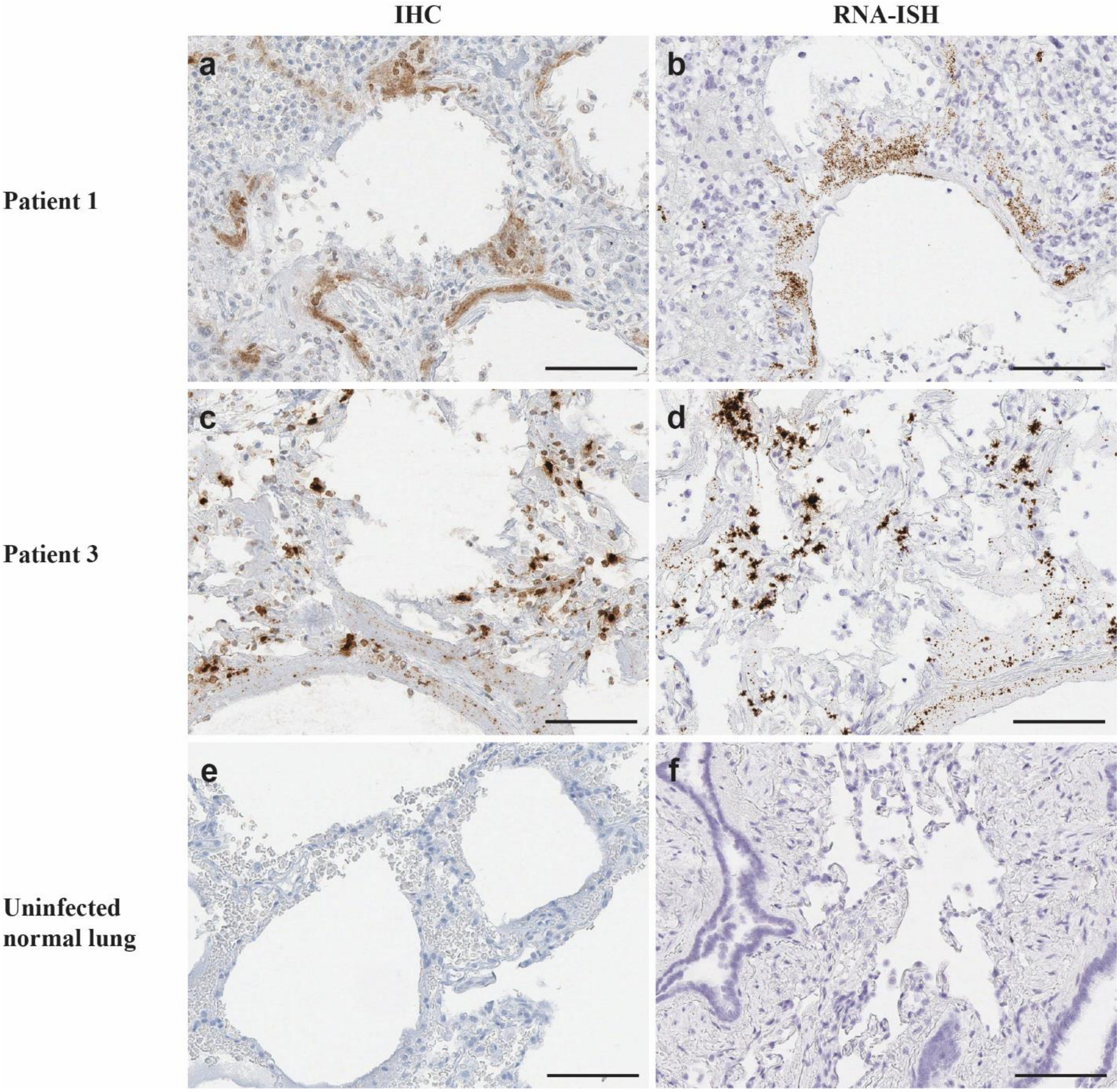

Supplementary Figure 3. ***ACE2*** **expression by RNA-ISH in pulmonary and non-pulmonary tissues.** *ACE2* expression was detected in **a**) respiratory epithelium of trachea, **b**) sub-epithelial bronchial sero-mucinous glands, **c**) lining bronchial epithelia cells (left) and a subset of alveolar epithelial cells (right), **d**) biliary epithelium of bile duct and portal tract in liver, **e**) prostatic acinar epithelium (inset shows high focal ACE2 expression), **f**) intra-follicular region in thyroid, **g**) within lymph node, **h**) colonic mucosal glands, and **i**) within myocardial cells and stromal/endothelial cells in myocardium. Scale bar=50µm. Inset scale bar=10µm.d

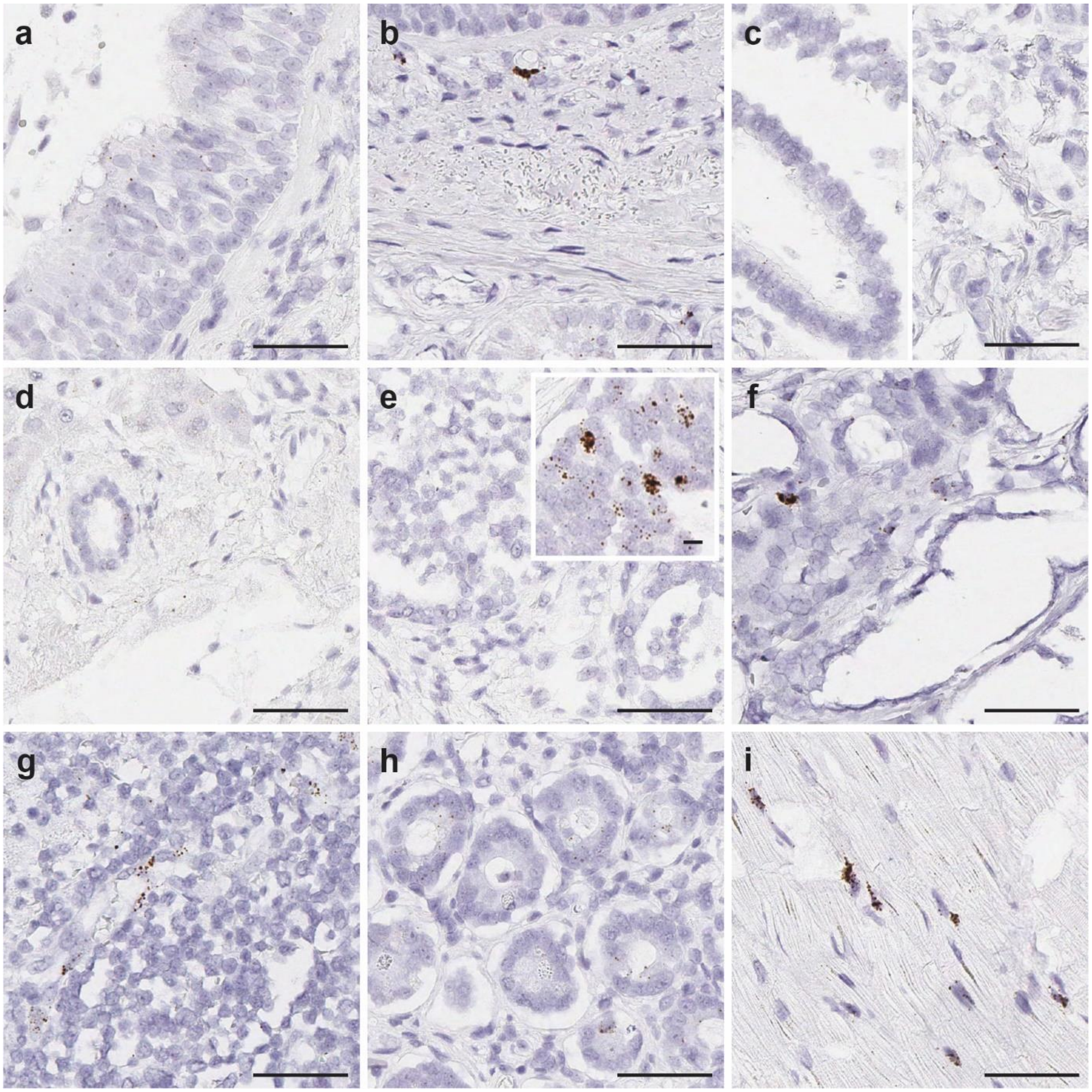

Supplementary Figure 4. *TMPRSS2* expression in pulmonary and non-pulmonary tissues. *TMPRSS2* expression was detected in **a**) respiratory epithelium of trachea, **b**) sub-epithelial bronchial sero-mucinous glands, **c**) bronchial epithelial cells, **d**) a sub-set of alveolar epithelial cells, **e**) prostatic acinar epithelium, **f**) liver parenchyma, **g**) both lining follicular epithelium and in intra-follicular region of thyroid, **h**) cortical renal tubules of kidney, and **i**) intestinal mucosal glands. Scale bar=50μm.

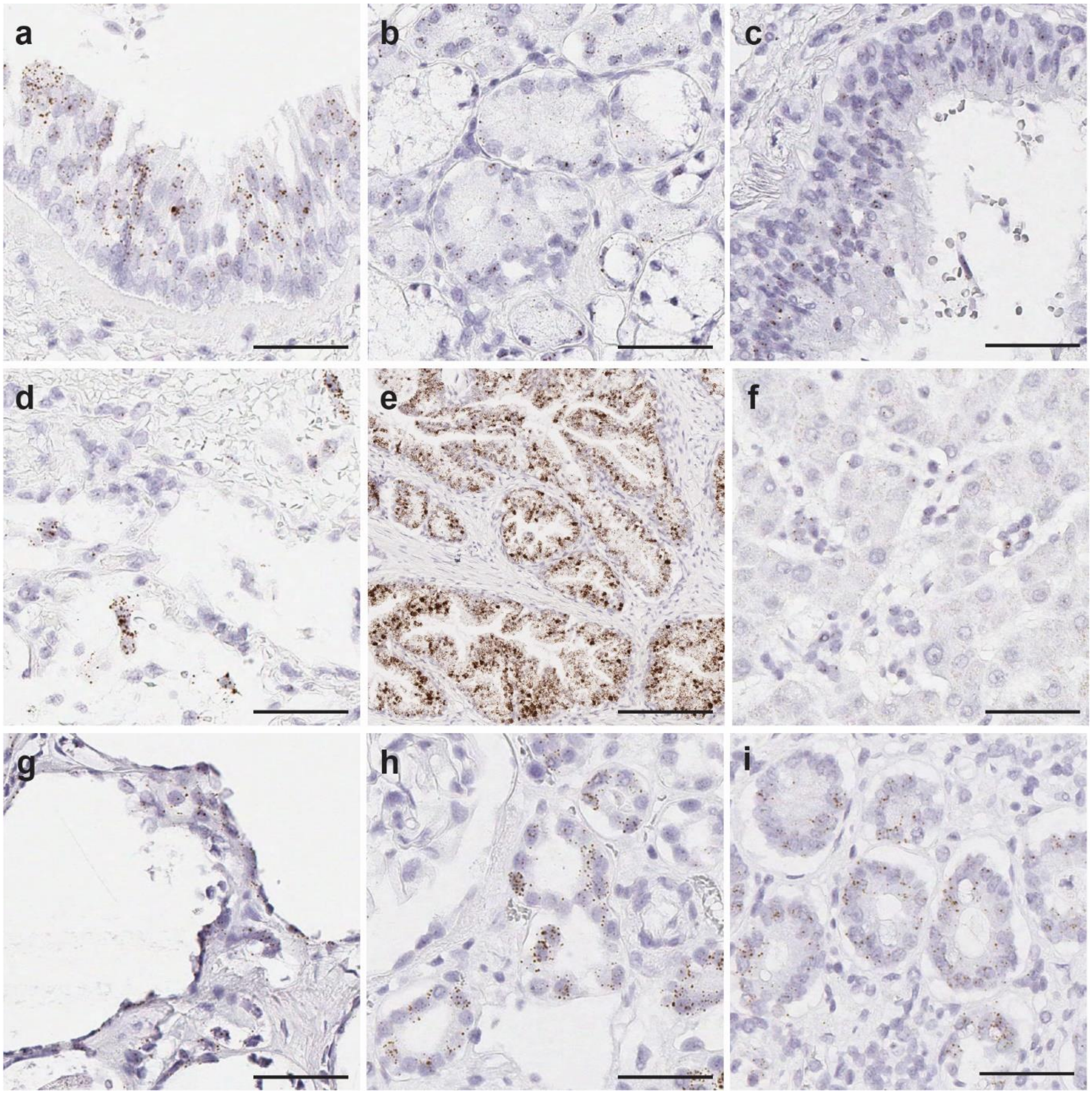

Supplementary Figure 5. ***AR* expression in pulmonary and non-pulmonary tissues.** *AR* expression was detected in **a**) respiratory epithelium of bronchus, **b**) sub-epithelial bronchial sero-mucinous glands, **c**) bronchial epithelial cells, **d**) a sub-set of alveolar epithelial cells, **e**) sub-epithelial tracheal sero-mucinous glands, **f)** prostatic acinar epithelium, **g**) both lining follicular epithelium and in intra-follicular region of thyroid, **h**) cortical glomerular and renal tubules of kidney, and **i**) liver parenchyma. Scale bar=50μm. Arrows point to RNA-ISH signals.

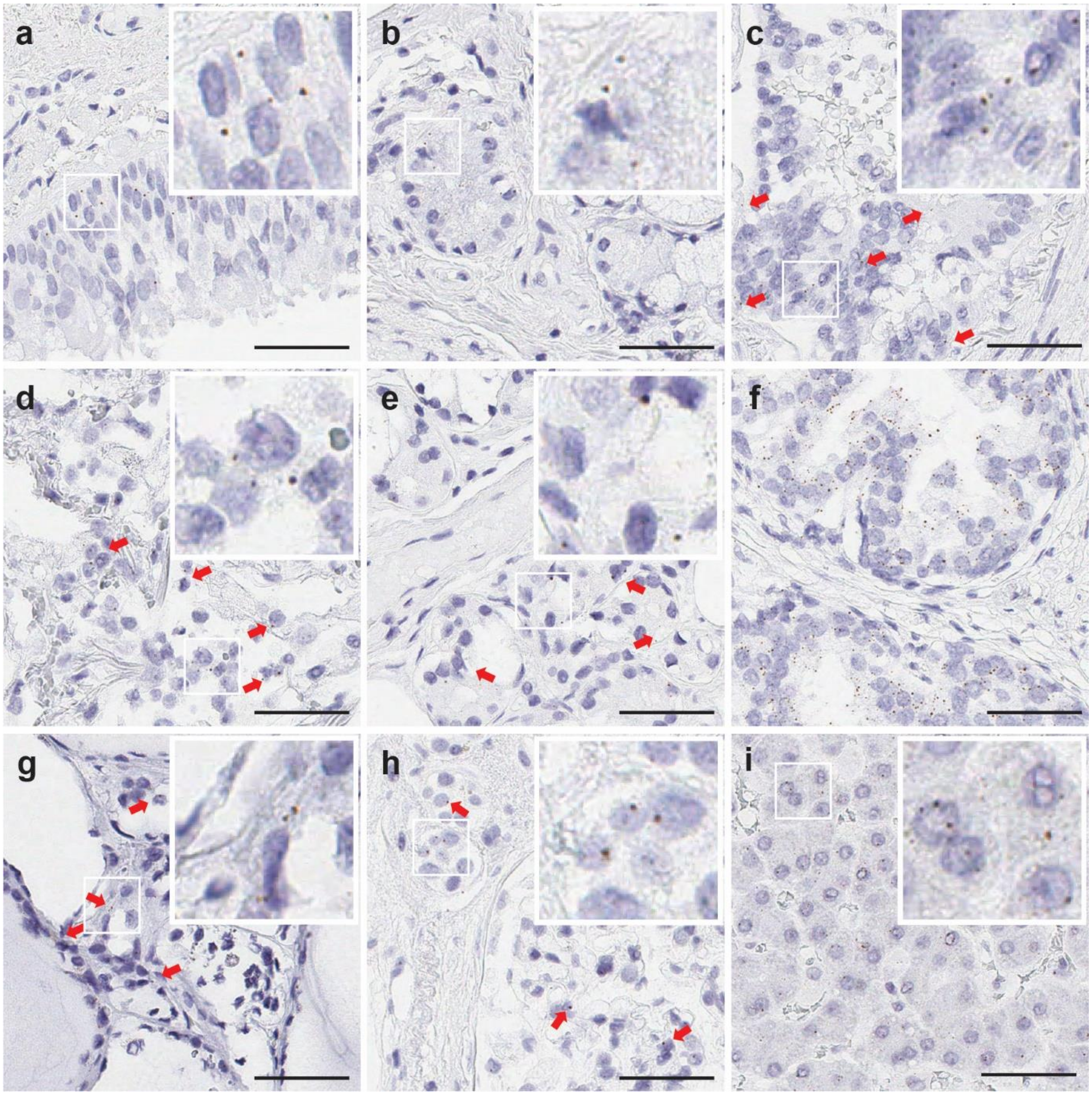

Supplement: Supplementary file 1 — Supplementary Information [file 43856_2021_25_MOESM1_ESM.pdf]
