# Supplementary material for: Characterization of SARS-CoV-2 and host entry factors distribution in a COVID-19 autopsy series
Source: Commun Med (Lond). 2021 Aug 23;1:24. doi: 10.1038/s43856-021-00025-z (PMC9053209; doi:10.1038/s43856-021-00025-z)
Supplement: Supplementary file 3 — Reporting Summary [file 43856_2021_25_MOESM3_ESM.pdf]

nature research

Corresponding author(s): Mehra Rohit

Last updated by author(s): Jun 1, 2021

Reporting Summary

Nature Research wishes to improve the reproducibility of the work that we publish. This form provides structure for consistency and transparency in reporting. For further information on Nature Research policies, see our Editorial Policies and the Editorial Policy Checklist.

Statistics

For all statistical analyses, confirm that the following items are present in the figure legend, table legend, main text, or Methods section.

n/a	Confirmed	
☐	☒	The exact sample size (*n*) for each experimental group/condition, given as a discrete number and unit of measurement
☐	☒	A statement on whether measurements were taken from distinct samples or whether the same sample was measured repeatedly
☒	☐	The statistical test(s) used AND whether they are one- or two-sided<br>*Only common tests should be described solely by name; describe more complex techniques in the Methods section.*
☒	☐	A description of all covariates tested
☒	☐	A description of any assumptions or corrections, such as tests of normality and adjustment for multiple comparisons
☒	☐	A full description of the statistical parameters including central tendency (e.g. means) or other basic estimates (e.g. regression coefficient) AND variation (e.g. standard deviation) or associated estimates of uncertainty (e.g. confidence intervals)
☒	☐	For null hypothesis testing, the test statistic (e.g. *F*, *t*, *r*) with confidence intervals, effect sizes, degrees of freedom and *P* value noted<br>*Give P values as exact values whenever suitable.*
☒	☐	For Bayesian analysis, information on the choice of priors and Markov chain Monte Carlo settings
☒	☐	For hierarchical and complex designs, identification of the appropriate level for tests and full reporting of outcomes
☒	☐	Estimates of effect sizes (e.g. Cohen's *d*, Pearson's *r*), indicating how they were calculated

Our web collection on statistics for biologists contains articles on many of the points above.

Software and code

Policy information about availability of computer code

Data collection	No software was used
Data analysis	Graphpad Prism 8 was used for making figure plot

For manuscripts utilizing custom algorithms or software that are central to the research but not yet described in published literature, software must be made available to editors and reviewers. We strongly encourage code deposition in a community repository (e.g. GitHub). See the Nature Research guidelines for submitting code & software for further information.

Data

Policy information about availability of data

All manuscripts must include a data availability statement. This statement should provide the following information, where applicable:

- Accession codes, unique identifiers, or web links for publicly available datasets
- A list of figures that have associated raw data
- A description of any restrictions on data availability

The datasets generated during and/or analysed during the current study are available from the corresponding author on reasonable request.

Field-specific reporting

Please select the one below that is the best fit for your research. If you are not sure, read the appropriate sections before making your selection.

☒ Life sciences ☐ Behavioural & social sciences ☐ Ecological, evolutionary & environmental sciences

For a reference copy of the document with all sections, see nature.com/documents/nr-reporting-summary-flat.pdf

Life sciences study design

All studies must disclose on these points even when the disclosure is negative.

Sample size	We collected FFPE tissue blocks from 16 different anatomical sites from 6 patients who died from COVID-19 disease with clinical autopsies performed. This current study provided a descriptive report of SARS-CoV-2 infection and host factor expression in the COVID-19 autopsy tissues.
Data exclusions	For RNA-ISH staining, the RNA quality was accessed using house keeping gene PPIB as positive control, the sample was excluded if PPIB staining is less than 1-3 dots per cell.
Replication	The reproducibility of the findings in this study was confirmed by biological replicates, including multiple tissue blocks of same and different anatomy sites for the same patient and tissues from different patients. The reproducibility was also confirmed by different experimental approaches as detailed in the manuscript
Randomization	The samples were block randomized and stained in multiple groups on different dates.
Blinding	During data evaluation, the patient and normal controls were coded and evaluated blindly by multiple researchers independently

Reporting for specific materials, systems and methods

We require information from authors about some types of materials, experimental systems and methods used in many studies. Here, indicate whether each material, system or method listed is relevant to your study. If you are not sure if a list item applies to your research, read the appropriate section before selecting a response.

Materials & experimental systems

n/a	Involved in the study
☐	☒ Antibodies
☒	☐ Eukaryotic cell lines
☒	☐ Palaeontology and archaeology
☒	☐ Animals and other organisms
☒	☐ Human research participants
☒	☐ Clinical data
☒	☐ Dual use research of concern

Methods

n/a	Involved in the study
☒	☐ ChIP-seq
☒	☐ Flow cytometry
☒	☐ MRI-based neuroimaging

Antibodies

Antibodies used	SARS-CoV-2 nucleocapsid IHC was performed using mouse monoclonal antibody (R&D system MAB10474) at 1:100 dilutions with heat-induced epitope retrieval with TRIS antigen retrieval buffer.
Validation	The antibody was optimized and validated using previously confirmed positive and negative clinical patient samples. The vendor also provided validation data (western blot and IHC) on the website (https://www.rndsystems.com/products/sars-cov-2-nucleocapsid-antibody-1035111_mab10474).

April 2020
